# Response to Letter to the Editor: "Evaluate the effects of curcumin on the prevention of atrial and ventricular arrhythmias and heart failure in patients with unstable angina"

**Published:** 2020

**Authors:** Mostafa Dastani, Asieh Karimani, Amir Hooshang Mohammadpour

**Affiliations:** 1 *Department of Cardiology, Faculty of Medicine, Mashhad University of Medical Sciences, Mashhad, Iran *; 2 *Department of Pharmacodynamics and Toxicology, School of Pharmacy, Mashhad University of Medical Sciences, Mashhad, Iran*; 3 *Department of Clinical Pharmacy, School of Pharmacy, Mashhad University of Medical Sciences, Mashhad, Iran *; 4 *Pharmaceutical Research Center, Institute of Pharmaceutical Technology, Mashhad University of Medical Sciences, Mashhad, Iran *

**Dear editor**

Regarding the duration of supplement therapy in our study which has been published in the Avicenna J Phytomed, 2019; 9(1): 1-9, “The effects of curcumin on the prevention of atrial and ventricular arrhythmias and heart failure in patients with unstable angina” (Dastani et al., 2019 ▶), some critical flaws has been mentioned. Primarily, we are grateful for the consideration of this article and we also appreciate the suggestions which have been very useful. Here, we will describe the most important point which was missed.

The duration of supplement therapy was about 5 days and all the related complications were measured after this time.

Acute coronary syndrome is a medical emergency (Switaj et al., 2017 ▶) that begins with tearing of atherosclerosis plaque in one of the coronary epicardial arteries and the thrombus placement on the atherosclerosis plaque (Bentzon et al., 2014 ▶). Inflammation plays a key role in rupturing the plaque and pathophysiology of acute coronary syndrome (Mulvihill et al., 2002 ▶). The chances of full vein closure increases when plaques tear off, and eventually an unstable angina can progress to a myocardial infarction (Willerson et al., 1991 ▶). All these happenings could be completed in 5 days. By inhibiting inflammation or other responsible factors during this period, the progression of unstable angina towards the myocardial infarction can be prevented. Therefore, the patients with no significant risk factor can find a stable condition within 5 days of hospital admission. Regarding the high role of inflammation during the first days of acute coronary syndromes and stabilization of the patients after that time, the study period of this study was 5 days.

## Conflict of interest

The authors have declared that there is no conflict of interest.
